# Comparative Analysis of Bacteremic and Non-bacteremic Sepsis: A Retrospective Study

**DOI:** 10.7759/cureus.76418

**Published:** 2024-12-26

**Authors:** Ryan Lee, Rawaa Al Rifaie, Keshab Subedi, Christian Coletti

**Affiliations:** 1 Emergency Medicine, Christiana Care Health System, Newark, USA; 2 Pulmonary and Critical Care Medicine, University of Maryland Medical Center, Baltimore, USA; 3 Biostatistics, Christiana Care Health System, Newark, USA

**Keywords:** bacteremia, critical care, emergency medicine, gram-negative bacteria, gram-positive bacteria, septic shock

## Abstract

Introduction: Sepsis remains a prevalent critical illness encountered in emergency departments and intensive care units (ICU), with culture-negative sepsis constituting 30-60% of cases. The effect of culture type on treatment and outcomes remains unclear, and conflicting evidence exists regarding disparities between Gram-positive and Gram-negative infections.

Objective: To further describe and compare characteristics and outcomes of culture-positive versus culture-negative sepsis.

Design, setting and participants: This retrospective cohort study included 1375 patients admitted to the ICU of a single tertiary care hospital between 2016 and 2019 with a diagnosis of sepsis or septic shock. Patients who did not meet the screening criteria, lacked drawn or documented cultures, or had documented non-bacterial infections, were excluded.

Main outcomes and measures: The primary outcome was disease severity and secondary outcomes included in-hospital mortality and duration of hospital and ICU stay. The principal and secondary exposure variables were blood culture status (positive vs. negative) and Gram staining (positive vs. negative), respectively.

Results: Overall, 943 patients (68.5%) were culture-negative and 432 (31.5%) were culture-positive. Gram-positive bacteria were isolated from 178 patients, Gram-negative bacteria from 199 patients, and both from 55 patients. Culture-positive patients demonstrated an almost two-fold higher likelihood of requiring vasopressors (adjusted odds ratio (OR): 1.98), a higher incidence of stress-dose steroid administration (adjusted OR, 1.68), and higher resuscitative fluid administration at six and 72 hours than culture-negative patients. No significant between-group differences emerged in the ICU or hospital length of stay, or mortality. No significant variations were observed when comparing Gram-positive and Gram-negative bacteremia.

Conclusion: Although significant differences in illness severity existed between blood culture-negative and blood culture-positive patients with sepsis, patient-oriented secondary outcomes did not exhibit significant between-group differences. These results indicate that clinicians should not be reassured by the lack of proven bacteremia in patients with suspected sepsis, given similar outcomes.

## Introduction

The global effect of sepsis is staggering, affecting more than 30 million individuals each year and potentially leading to six million fatalities [1]. In 2016, a redefined classification of sepsis, labeled Sepsis-3, was introduced to better describe it as an infection accompanied by organ dysfunction. This revision aimed to provide a more nuanced understanding of the condition [1]. Despite decades of dedicated clinical trials to unravel the complexities of sepsis, mortality rates remain at approximately 20% [1-3]. The challenge in reducing sepsis-related mortality rates underscores the need to improve our understanding of the pathophysiology of the disease and develop targeted management strategies.

A pivotal moment in sepsis care emerged in 2001 with the publication of the Rivers Trial which advocated the advantages of early goal-directed therapy (EGDT) [1]. However, a series of randomized controlled trials conducted a decade later failed to demonstrate any discernible benefit of EGDT over standard protocolized care [1-3]. Recent literature has attempted to identify the specific aspects of sepsis that may indicate higher mortality rates, yielding varied results. Evidence suggests that higher fluid resuscitation volumes and a positive fluid balance may correlate with higher mortality rates in cases of septic shock [1-10]. Similarly, an influx of recent studies examining the relationships between culture data, the organism responsible for infection, and the source of infection, has occurred with mixed conclusions regarding their effects on mortality [1-7]. Classic teaching has asserted that Gram-negative infections tend to increase disease severity and mortality. However, recent trials have challenged this long-standing belief [1-4,11,12].

Determining the relationship between culture data and disease severity has numerous implications, influencing treatment approaches, and offering improved prognostic insights. It may not only shape antibiotic choices, but also determine the aggressiveness of treatment, including earlier intervention with source control and the initiation of vasopressors and steroids. The primary study objective was to evaluate disease severity (assessed through vasopressor need, ventilatory support, and intravenous fluid (IVF) requirements) in patients admitted to the intensive care unit (ICU) with a diagnosis of sepsis, and to compare those with blood culture-positive (bacteremic) and blood culture-negative (non-bacteremic) sepsis. Secondary outcomes, including ICU stay, hospital length of stay (LOS), and mortality rates, were also compared between the two groups. Furthermore, a predefined subgroup analysis was conducted on culture-positive patients to stratify outcomes based on Gram staining results (positive vs. negative). We hypothesized that bacteremic sepsis would exhibit higher severity than non-bacteremic sepsis, correlating directly with the need for vasopressors, ventilatory support, and IVF requirements. In-hospital mortality and ICU and hospital LOS were expected to correlate directly with illness severity. Additionally, we hypothesized that Gram-negative infections would manifest higher disease severity and mortality than Gram-positive infections.

The article was previously presented as a meeting abstract at the ATS International Conference on May 17, 2021.

## Materials and methods

Study design and setting

This retrospective cohort study was conducted in the Medical Intensive Care Unit at Christiana Care Hospital, a prominent healthcare institution and one of the largest healthcare providers in the mid-Atlantic region. Functioning as a community academic hospital, it boasts a Level 1 trauma center catering to Delaware, Pennsylvania, Maryland, and New Jersey. The hospital itself is an 825-bed tertiary care center that encompasses four adult ICUs with a combined capacity of approximately 80 beds.

The study included patients aged ≥18 years admitted from the emergency department (ED) to the ICU with an International Classification of Diseases, Ninth Revision (ICD-9) or ICD-10 diagnosis code of sepsis, severe sepsis, or septic shock (A41.9, R65.21, 995.91, 995.92, and 785.52) between January 1, 2016, and December 31, 2019. Individuals without a documented blood culture, with a documented fungal infection, who left against medical advice, and who did not receive antibiotics at 72 hours or were not receiving them at the time of death, were excluded. The rationale for the final exclusion criteria was to eliminate patients for whom a bacterial infection was not suspected to be an ongoing diagnosis following admission, thereby focusing on cases in which sepsis and related conditions were more likely.

This study was approved by the Christiana Care Institutional Review Board (IRB00000480 CCC#40039, approved on 03/26/2020).

Data collection

Data were retrieved from the electronic health record (EHR) system of Christiana Care Hospital and included patient demographic details such as age, race, and ethnicity. In-hospital mortality, hospital and ICU LOS, the volume of fluid ordered in the first six hours and first 72 hours (distinct from the actual fluid volume administered but not documented), use of vasopressors, steroids, and the need for mechanical ventilation were also collected. Additionally, the Charlson comorbidity scores for conditions such as congestive heart failure, liver disease, chronic lung disease, and renal failure were obtained. Laboratory results from blood cultures were used to categorize patients into culture-positive and culture-negative groups, as well as into Gram-positive and Gram-negative groups.

The principal exposure variable analyzed was blood culture status (positive vs. negative). Culture positivity was defined as having at least one blood culture with bacterial growth, while excluding other cultures such as urine, sputum, endotracheal, and CSF. The secondary exposure variable was Gram staining (positive vs. negative) to evaluate outcomes among culture-positive patients.

The primary outcome was disease severity as measured by IVF volume in the first six and 72 hours after presentation, along with the use of vasopressors (yes/no), steroids (yes/no), and mechanical ventilation (yes/no). The secondary outcomes included in-hospital mortality and duration of hospital and ICU stay. The in-hospital mortality status was determined by discharge disposition. Patient transfers to other hospitals were infrequent and the effects were negligible. Hospital LOS was calculated as the time from admission to the ED to discharge from the hospital, whereas the ICU LOS was determined as the duration from admission to the ICU to discharge from the ICU.

Statistical analysis

The demographic and clinical characteristics of the overall study population and the populations stratified by culture (positive vs. negative) and Gram status (positive vs. negative) were summarized using mean and standard deviation for continuous variables, and count and percentage for categorical variables. Comparisons between the culture-positive and culture-negative groups, as well as between the Gram-positive and Gram-negative groups, were conducted using Wilcoxon rank sum tests and chi-squared tests.

Multivariate log-normal regression models were then employed to estimate the adjusted effect of culture on the total IVF volumes in the first six and 72 hours. A log transformation was applied to address the high right skewness in the distribution of fluid volumes. The covariates included in these models were culture (positive vs. negative), Charlson comorbidity score, sex, race, ethnicity, history of renal failure, congestive heart failure, chronic lung disease, and body weight. These covariates were preselected, and model-based variable selection was not employed. For easier interpretation, the coefficient estimates from the log-normal models were exponentiated, with the exponentiated coefficients representing the ratio of the expected volume among patients with and without the specified factor. Additionally, multivariate logistic regression models were fitted for mortality, and vasopressor, ventilator, and steroid use, while adjusting for the same set of variables as those in the regression models for fluid volume.

All statistical analyses were conducted using Statistical Analysis System (SAS) 9.4 (SAS Institute Inc., Cary, NC, USA). P value was defined as <0.05. The requirement for informed consent was waived due to the anonymous nature of our data. The study was conducted in accordance with both internal institutional ethical standards and the Helsinki Declaration of 1975.

## Results

Baseline characteristics

We initially identified 1534 patients who met the inclusion criteria. After excluding patients without a blood culture drawn (N=45), those with a documented fungal infection (N=5), those who left against medical advice (N=10), and those who did not receive antibiotics within 72 hours of admission or were not receiving antibiotics at the time of death (N=99), the final analysis included 1375 patients, with 943 (68.5%) classified as culture-negative and 432 (31.5%) as culture-positive.

The mean age of the overall study population was 65.5 ± 15.5 years, with 50.2% male, and 75.0% White patients. Table 1 outlines the baseline characteristics of all patients. Comparatively, culture-positive patients were more likely to have chronic lung disease (52% vs. 44%) than were culture-negative patients, whereas other baseline characteristics were similar. In the culture-positive group, patients with Gram-negative cultures tended to be younger than those with Gram-positive cultures. However, the distributions of sex, race, ethnicity, and comorbidities were comparable between Gram-negative and Gram-positive patients.

**Table 1:** Demographic and clinical characteristics of the study population. The values are counts and percentages unless otherwise noted. SD: standard deviation; IQR: interquartile range; +ve: positive; -ve: negative

	Culture			Gram		
Variable	All (n=1375)	Negative (n=943)	Positive (n=432)	Both (+ve/-ve) (n=55)	Negative (n=199)	Positive (n=178)
Age in years, Mean (± SD)	65.5 (15.5)	65.5 (15.3)	65.7 (15.9)	61.3 (16.3)	68.4 (15.3)	64.2 (16.1)
Sex, n (%)						
Female	685 (49.8)	483 (51.2)	202 (46.8)	24 (43.6)	94 (47.2)	84 (47.2)
Male	690 (50.2)	460 (48.8)	230 (53.2)	31 (56.4)	105 (52.8)	94 (52.8)
Race, n (%)						
White	1031 (75.0)	696 (73.8)	335 (77.6)	39 (70.9)	155 (77.9)	141 (79.2)
Black	263 (19.1)	188 (19.9)	75 (17.4)	14 (25.5)	34 (17.1)	27 (15.2)
Other	81 (5.9)	59 (6.3)	22 (5.1)	2 (3.6)	10 (5.0)	10 (5.6)
Ethnicity, n (%)						
Hispanic or Latino	53 (3.9)	35 (3.7)	18 (4.2)	3 (5.5)	7 (3.5)	8 (4.5)
Non-Hispanic or Latino	1290 (93.8)	887 (94.1)	403 (93.3)	50 (90.9)	189 (94.8)	164 (92.1)
Unknown	32 (2.3)	21 (2.2)	11 (2.6)	2 (3.6)	3 (1.5)	6 (3.4)
Body weight in kg, Mean ± SD	83.3 (27.4)	83.4 (28.5)	82.9 (24.8)	84.0 (28.6)	80.6 (21.9)	85.1 (26.4)
Charlson score, Median (IQR)	4 (2, 7)	4 (2, 7)	4 (2, 7)	5 (2, 9)	4 (2, 7)	3 (1, 7)
History of renal failure, n (%)	421 (30.6)	290 (30.8)	131 (30.3)	15 (27.27)	58 (29.2)	58 (32.6)
History of congestive heart failure, n (%)	471 (34.3)	335 (35.5)	136 (31.5)	16 (29.1)	63 (31.7)	57 (32.0)
History of liver disease, n (%)	67 (4.9)	40 (4.2)	27 (6.3)	3 (5.5)	11 (5.5)	13 (7.3)
History of chronic lung disease, n (%)	684 (49.8)	492 (52.2)	192 (44.4)	26 (47.3)	82 (41.2)	84 (47.2)

Outcomes by culture status

Table 2 presents a bivariate comparison of the outcomes based on the culture status. Notably, culture-positive patients received significantly higher fluid volumes than culture-negative patients at both six and 72 hours. Culture-positive patients were more likely to require vasopressor support (47.9% vs. 31.3%, P<0.001) and steroid administration than culture-negative patients (44.9% vs. 32.6%, P<0.001). Conversely, culture-negative patients had a higher incidence of requiring ventilator support than culture-positive patients (72.9% vs. 61.8%; P<0.001). No significant differences were observed in ICU and hospital LOS between culture-positive and culture-negative patients. Additionally, in-hospital mortality rates were comparable between the two groups.

**Table 2:** Bivariate comparison of the outcomes by culture status. The p-values are based on Wilcoxon rank sum tests for the first six hours of fluid volume, first 72 hours of fluid volume, vasopressor duration, ICU length of stay and hospital length of stay, and Chi-squared test for vasopressor use, steroid use, ventilation use, and mortality. IQR: interquartile range; ICU: intensive care unit

Variable	All (n=1375)	Negative (n=943)	Positive (n=432)	P-value
First 6 Hours Fluid Volume, mL (median, IQR)	2000 (1000, 3000)	2000 (1000, 3000)	2000 (1000, 3000)	<0.001
First 72 Hours Fluid Volume, mL (median, IQR)	3000 (2000, 5000)	3000 (2000, 4500)	4000 (2500, 5000)	<0.001
Vasopressor Use, n (%)	502 (36.5)	295 (31.3)	207 (47.9)	<0.001
Vasopressor Duration, Hours (median, IQR)	30.5 (24.0, 45.0)	28.0 (24.0, 43.0)	33.0 (24.0, 49.0)	0.268
Steroid Use, n (%)	501 (36.4)	307 (32.6)	194 (44.9)	<0.001
Ventilation Use, n (%)	955 (69.5)	688 (72.9)	267 (61.8)	<0.001
ICU Length of Stay, Hours (median, IQR)	69.0 (40.0, 137.0)	72.0 (40.0, 147.0)	64.5 (40.0, 119.5)	0.059
Hospital Length of Stay, Days (median, IQR)	9.17 (5.13, 15.63)	9.29 (5.13, 15.58)	9.04 (5.02, 15.81)	0.649
Mortality, n (%)	327 (23.8)	215 (22.8)	112 (25.9)	0.206

The outcomes of the multivariate model for fluid administration at six hours and 72 hours are detailed in Tables 3-4. Relative to culture-negative patients, those who were culture-positive received higher fluid volumes within the first six hours, with an adjusted mean increase of 26% (CI: 18-35%, P<0.001). Similarly, the multivariate model indicated that the fluid volume administered in the first 72 hours was 16% higher (CI: 8-24%) in culture-positive than in culture-negative patients. The associations between patient blood culture status (positive vs. negative) and vasopressor administration, steroid administration, and ventilation use are presented in Tables 5-7. When adjusting for demographic and clinical characteristics, the odds of patients receiving vasopressors were 1.98 times higher (CI: 1.57, 2.51) among culture-positive patients than among culture-negative patients. Likewise, culture-positive status was associated with higher odds of steroid use by a factor of 1.68 (CI: 1.33, 2.12) than for culture-negative status. Conversely, culture-positive status was linked to lower odds of ventilator use by a factor of 0.61 (CI: 0.48, 0.78) than for culture-negative status.

**Table 3:** Results from the log-normal regression model of total fluid volume in the first six hours. Note: For ease of interpretation, the coefficients from the log-normal model are exponentiated. The exponentiated coefficients (e β) can be interpreted as a ratio of expected fluid volume for patients at a given label of a parameter to the reference label of the parameter. For example, the expected fluid volume in the first six hours for patients with culture-positive is 1.26 times the expected fluid volume in the first six hours for patients with culture-negative. However, there is no significant difference in the expected fluid volume between gram-negative and gram positive-cultures. P-values are based on the log-normal regression model.

	Culture		Gram	
Parameters	e β (95% CI)	P-value	e β (95% CI)	P-value
Culture Positive vs Negative	1.26 (1.18, 1.35)	<0.001		
Gram: Both vs Negative			1.01 (0.86, 1.19)	0.885
Gram Positive vs Negative			1.03 (0.92, 1.15)	0.649
Age	1.00 (0.99, 1.00)	0.002	0.99 (0.99, 1.00)	0.002
Charlson Comorbidity Score	1.00 (0.99, 1.02)	0.387	1.01 (0.99, 1.03)	0.187
Gender: Female vs Male	0.94 (0.88, 1.01)	0.084	0.93 (0.83, 1.03)	0.168
Race: Black vs White	0.92 (0.84, 1.01)	0.073	0.82 (0.70, 0.97)	0.017
Race: Other vs White	0.98 (0.84, 1.15)	0.832	0.92 (0.68, 1.23)	0.558
Ethnicity: Hispanic or Latino (HL) vs Non-Hispanic or Latino (NHL)	1.02 (0.86, 1.21)	0.814	0.83 (0.61, 1.15)	0.269
Ethnicity: Unknown vs NHL	1.01 (0.80, 1.27)	0.937	1.10 (0.79, 1.53)	0.574
History of Renal Failure	0.89 (0.81, 0.97)	0.011	0.80 (0.69, 0.93)	0.003
History of Congestive Heart Failure	0.87 (0.80, 0.96)	0.003	0.94 (0.82, 1.09)	0.434
History of Chronic Lung Disease	1.00 (0.93, 1.08)	0.989	0.94 (0.83, 1.06)	0.324
Weight	1.00 (1.00, 1.00)	0.071	1.00 (1.00, 1.00)	0.072

**Table 4:** Results from the log-normal regression model of total fluid volume in first 72 hours. Note: For ease of interpretation, the coefficients from the log-normal model are exponentiated. The exponentiated coefficients (e β) can be interpreted as a ratio of expected fluid volume for patients at a given label of a parameter to the reference label of the parameter. For example, the expected fluid volume in the first 72 hours for patients with culture-positive is 1.16 times the expected fluid volume in the first 72 hours for patients with culture-negative. However, there is no significant difference in the expected fluid volume between gram-negative and gram positive-cultures. P-values are based on the log-normal regression model.

	Culture (Positive vs Negative)		Gram	
Parameters	e β (95% CI)	P-value	e β (95% CI)	P-value
Culture Positive vs Negative	1.16 (1.08, 1.24)	<0.001		
Gram: Both vs Negative			1.11 (0.94, 1.32)	0.23
Gram Positive vs Negative			1.02 (0.90, 1.15)	0.803
Age	1.00 (0.99, 1.00)	0.006	1.00 (0.99, 1.00)	0.288
Charlson Comorbidity Score	0.99 (0.98, 1.00)	0.09	0.99 (0.97, 1.01)	0.256
Gender: Female vs Male	0.98 (0.92, 1.05)	0.641	1.03 (0.92, 1.15)	0.65
Race: Black vs White	0.92 (0.84, 1.01)	0.069	0.79 (0.66, 0.94)	0.007
Race: Other vs White	1.03 (0.89, 1.19)	0.712	1.18 (0.90, 1.54)	0.233
Ethnicity: Hispanic or Latino (HL) vs Non-Hispanic or Latino (NHL)	1.04 (0.88, 1.23)	0.621	0.94 (0.70, 1.26)	0.678
Ethnicity: Unknown vs NHL	1.19 (0.93, 1.51)	0.173	1.10 (0.74, 1.64)	0.631
History of Renal Failure	0.95 (0.86, 1.04)	0.255	0.88 (0.75, 1.04)	0.136
History of Congestive Heart Failure	0.98 (0.89, 1.07)	0.584	1.00 (0.86, 1.16)	0.996
History of Chronic Lung Disease	0.95 (0.88, 1.03)	0.193	0.93 (0.81, 1.06)	0.264
Weight	1.00 (1.00, 1.00)	0.834	1.00 (1.00, 1.00)	0.928

**Table 5:** Odds ratio (OR) estimates from the logistic regression model of vasopressor use.

	Culture	Gram
Effect	Adjusted OR (95% CI)	Adjusted OR (95% CI)
Culture Positive vs Negative	1.98 (1.57, 2.51)	
Gram: Both vs Negative		1.04 (0.57, 1.92)
Gram Positive vs Negative		0.70 (0.46, 1.05)
Age	1.00 (0.99, 1.00)	1.00 (0.98, 1.01)
Gender: Female vs Male	0.85 (0.68, 1.060	0.82 (0.56, 1.20)
Race: Black vs White	1.06 (0.79, 1.42)	0.90 (0.53, 1.52)
Race: Other vs White	0.79 (0.46, 1.34)	1.26 (0.46, 3.45)
Ethnicity: Hispanic or Latino (HL) vs Non-Hispanic or Latino (NHL)	0.76 (0.40, 1.46)	0.56 (0.19, 1.70)
Ethnicity: Unknown vs NHL	2.93 (1.38, 6.23)	1.11 (0.31, 3.93)
History of Renal Failure	1.02 (0.78, 1.34)	1.00 (0.63, 1.59)
History of Congestive Heart Failure	0.87 (0.66, 1.14)	0.74 (0.45, 1.21)
History of Chronic Lung Disease	0.83 (0.65, 1.06)	0.98 (0.64, 1.49)

**Table 6:** Odds ratio (OR) estimates from the logistic regression model of steroid use.

	Culture	Gram
Effect	Adjusted OR (95% CI)	Adjusted OR (95% CI)
Culture Positive vs Negative	1.68 (1.33, 2.12)	
Gram: Both vs Negative		1.52 (0.82, 2.81)
Gram Positive vs Negative		0.91 (0.60, 1.38)
Age	1.00 (0.99, 1.01)	1.01 (0.99, 1.02)
Gender: Female vs Male	1.02 (0.82, 1.28)	0.84 (0.57, 1.23)
Race: Black vs White	1.11 (0.83, 1.48)	1.05 (0.62, 1.78)
Race: Other vs White	0.86 (0.51, 1.45)	1.72 (0.62, 4.73)
Ethnicity: Hispanic or Latino (HL) vs Non-Hispanic or Latino (NHL)	1.03 (0.56, 1.92)	0.76 (0.25, 2.28)
Ethnicity: Unknown vs NHL	1.25 (0.60, 2.62)	0.80 (0.22, 2.91)
History of Renal Failure	1.34 (1.03, 1.75)	1.09 (0.69, 1.74)
History of Congestive Heart Failure	0.79 (0.60, 1.04)	0.61 (0.37, 1.00)
History of Chronic Lung Disease	0.96 (0.76, 1.23)	1.24 (0.81, 1.90)

**Table 7:** Odds ratio (OR) estimates from the logistic regression model of ventilation use.

	Culture	Gram
Effect	Adjusted OR (95% CI)	Adjusted OR (95% CI)
Culture Positive vs Negative	0.61 (0.48, 0.78)	
Gram: Both vs Negative		1.77 (0.92, 3.40)
Gram Positive vs Negative		2.00 (1.30, 3.10)
Age	0.99 (0.98, 1.00)	0.99 (0.98, 1.01)
Gender: Female vs Male	0.81 (0.64, 1.02)	0.81 (0.54, 1.21)
Race: Black vs White	1.83 (1.31, 2.55)	1.83 (1.03, 3.25)
Race: Other vs White	0.78 (0.47, 1.32)	1.33 (0.45, 3.940
Ethnicity: Hispanic or Latino (HL) vs Non-Hispanic or Latino (NHL)	1.02 (0.54, 1.92)	0.62 (0.20, 1.89)
Ethnicity: Unknown vs NHL	3.04 (1.13, 8.18)	2.43 (0.48, 12.16)
History of Renal Failure	0.85 (0.64, 1.12)	0.68 (0.42, 1.10)
History of Congestive Heart Failure	1.09 (0.82, 1.45)	1.05 (0.63, 1.75)
History of Chronic Lung Disease	1.36 (1.05, 1.75)	1.44 (0.92, 2.270)

Outcomes by gram staining status

Table 8 provides a bivariate comparison of outcomes based on the Gram staining status. Within the culture-positive group, fluid administration and vasopressor use did not show significant variation between Gram-positive and Gram-negative infections. However, Gram-positive bacteremia was associated with significantly higher rates of ventilator support than Gram-negative infections in culture-positive patients (69.7% vs. 52.8%, P=0.002). Additionally, culture-positive patients with Gram-positive bacteremia had a significantly longer ICU stay than those with Gram-negative infections (median 10.25 days vs. 7.71 days, P=0.003). Mortality did not vary significantly between the groups.

**Table 8:** Bivariate comparison of the outcomes by Gram status. The p-values are based on Wilcoxon rank sum tests for the first six hours of fluid volume, first 72 hours of fluid volume, vasopressor duration, ICU length of stay and hospital length of stay, and Chi-squared test for vasopressor use, steroid use, ventilation use, and mortality. IQR: interquartile range; ICU: intensive care unit; +ve: positive; -ve: negative

Variable	Both (+ve/-ve) (n=55)	Negative (n=199)	Positive (n=178)	P-value
First 6 hours Fluid Volume, mL (median, IQR)	2250 (2000, 3000)	2000 (1000, 3000)	2000 (1000, 3000)	0.827
First 72 hours Fluid Volume, mL (median, IQR)	4000 (2500, 5000)	4000 (3000, 5000)	3750 (2250, 5000)	0.911
Vasopressor Use, n (%)	29 (52.7)	102 (51.3)	76 (42.7)	0.188
Vasopressor Duration, Hours (median, IQR)	24.0 (18.0, 42.0)	33.5 (20.0, 50.0)	35.5 (24.0, 49.0)	0.218
Steroid Use, n (%)	30 (54.6)	89 (44.7)	75 (42.1)	0.269
Ventilation Use, n (%)	38 (69.1)	105 (52.8)	124 (69.7)	0.002
ICU Length of Stay, Hours (median, IQR)	83 (45, 154)	62 (37, 104)	63 (40, 137)	0.093
Hospital Length of Stay, Days (median, IQR)	10.96 (6.92, 23.96)	7.71 (4.88, 13.21)	10.25 (4.92, 17.92)	0.003
Mortality, n (%)	11 (20.00)	49 (24.62)	52 (29.21)	0.336

As in the "outcomes by culture status" section above, the outcomes of the multivariate model of fluid administration at six and 72 hours for Gram staining status are presented in Tables 3-4. No significant difference was observed in fluid administration between the Gram-positive and Gram-negative groups or for any controlled confounders. The associations between patient Gram staining status (positive vs. negative) and vasopressor administration, steroid administration, and ventilation use are detailed in Tables 5-7, respectively. No notable difference was observed in vasopressor or steroid use between the Gram-positive and Gram-negative groups or for any controlled confounders, except in patients with a history of renal failure who were more likely to receive steroids. However, when comparing Gram-positive and Gram-negative data based on the odds ratio for ventilation use, patients with Gram-positive infections were 2.0 times more likely (CI: 1.3-3.1) to require ventilation use than Gram-negative patients.

## Discussion

Few studies have directly compared blood culture-positive and culture-negative sepsis and septic shock. The primary findings of this study indicated that patients with blood culture-positive sepsis had a higher disease severity than those with blood culture-negative sepsis, as indicated by the higher volume of resuscitative fluid received and their higher likelihood of requiring vasopressor and glucocorticoid support. However, there were no significant differences in ICU and hospital LOS or rate of in-hospital mortality between the two groups.

Notably, this analysis showed a higher rate of culture-negative sepsis than that in many previously reported studies [11-16]. This difference could be attributed to our definition, which was limited to those with positive blood culture growth, as opposed to any other culture medium (urine, sputum, cerebrospinal fluid, peritoneal fluid, etc.). Nevertheless, our study demonstrated no significant difference in in-hospital mortality between the two groups, a finding consistent with the pan-European Sepsis Occurrence in Acutely Ill Patients (SOAP) study [17]. However, this finding differs from that of a recent study by Phua et al. in which culture-negative patients had significantly less disease severity and in-hospital mortality [14].

Although aggressive fluid resuscitation became a hallmark of septic shock in the early 2000s, recent studies have indicated a clear correlation between higher fluid volumes and hospital morbidity and mortality [7-10,18-23]. Our study demonstrated a statistically significant difference in the volumes of fluid administered to patients with culture-positive versus culture-negative sepsis. Although there was a trend towards higher mortality in culture-positive patients than in culture-negative patients, the difference was not statistically significant. Interestingly, vasopressor duration was longer in the culture-negative group compared to the culture-positive group, while ICU LOS was actually longer in the culture-negative group compared to the culture-positive group. This may be due to a non-statistically significant higher mortality in the culture-positive group decreasing the ICU and hospital LOS. Nonetheless, given the similar mortality rates, these data indicate that clinicians should not necessarily be reassured by a lack of culture growth when sepsis or septic shock is suspected. This finding likely reflects both the unaccounted-for variables affecting culture growth (amount of blood sampled, prior administration of antimicrobials, etc.) and the understanding that factors influencing mortality extend beyond the specific infectious organism. It may be more related to a combination of bacterial burden, underlying host immune defenses, preexisting conditions, and time to appropriate intervention. Although we did not specifically investigate these parameters, previous studies support this notion [1,13,24].

Our data showed no difference in mortality between Gram-positive and Gram-negative bacterial infections. However, a significantly higher rate of mechanical ventilatory support was observed in cases of Gram-positive sepsis than in Gram-negative sepsis. This difference is likely attributable to severe community-acquired pneumonia, primarily caused by Gram-positive bacteria, including streptococcal species, and post-viral staph pneumonia, which often necessitate intubation and mechanical ventilation. Conversely, Gram-negative pneumonia caused by bacteria such as *Klebsiella* and *Pseudomonas* is more commonly associated with ventilator-associated pneumonia and tends to occur predominantly in previously hospitalized patients who were not included in this study.

Our findings strongly suggest a higher illness severity in culture-positive patients than in those with culture-negative sepsis. Several hypotheses may explain the observed differences in disease severity, with one possible explanation being the inflammatory state at the microbiological level. The higher severity associated with culture-positive bacteremia could stem from direct endothelial damage, a higher bacteremic load, and an immune/systemic inflammatory response syndrome (SIRS) response, in contrast to the potentially milder effect of a lower bacterial burden in culture-negative sepsis [25-27]. Additionally, culture-negative patients may suffer from viremia or fungemia, potentially influencing disease severity [28,29]. The potential variability in the sensitivity/specificity of culture data due to non-standardized collection methods (such as exact blood volume in the testing tube, timing of antibiotic administration before sampling, and culture media) could also affect the sensitivity of the results [30]. Nonetheless, multiple factors could be at play, making this a promising area for future study, particularly novel collection and sampling methods, which may yield both higher sensitivity and specificity and allow for more targeted interventions.

Our study had several key limitations. Due to its retrospective nature, causation could not be established; however, the correlations observed can serve as a foundation for hypothesis generation in future prospective randomized controlled trials. Similarly, the trends towards better outcomes in culture-negative patients, while not statistically significant, may be explained by the underpowering of the patient population, as key markers of disease severity were significantly lower in this group. Additionally, the patient data is inevitably incomplete and may include interventions performed by prehospital providers that may not have been communicated or documented and the inherent measurement bias in documented versus effectively delivered interventions, particularly with regard to the volume of IVFs administered.

Moreover, our definition of bacteremic/culture-positive findings was confined to blood cultures. Some patients included in the culture-negative group may have had non-bacterial sepsis or non-infectious disease processes, although we believe our exclusion criteria accounted for most of these cases and therefore controlled for these confounding variables. Furthermore, while our rate of culture-negative sepsis is higher than that in some previously published studies [11-16], this is likely attributable to our definition of culture-positive sepsis, which only included those with positive blood cultures (excluding, for example, sputum, endotracheal, and urine). It is unclear whether the presence of other blood culture-negative groups that had other positive cultures, such as sputum, CSF, or urine, would have affected the patient outcomes in this study. Another limitation was the lack of control over appropriate antibiotic coverage or time to antibiotic administration. Patients who received antibiotics before cultures were drawn may have transitioned from a potentially culture-positive to a culture-negative group. Additionally, patient care was at the discretion of the ED and ICU providers. This involved decision-making regarding the volume of fluid resuscitation to be administered before initiating vasopressors and choices regarding empiric antibiotic coverage not being standardized across providers or departments. Finally, the findings of this study may not be broadly applicable to all ICU patients, including those admitted from the floor or after surgery, as we exclusively considered patients with sepsis admitted directly from the ED to the ICU.

## Conclusions

This single-center retrospective cohort study revealed no statistically significant differences in hospital or ICU LOS, or in-hospital mortality rates between patients with culture-positive and culture-negative sepsis. Despite significant variations in illness severity, as observed in IVF resuscitation, vasopressor use, and steroid requirements, when comparing culture-positive and culture-negative patients, no such differences translated into measurable effects on clinical outcomes. This implies that the factors influencing mortality likely extend beyond the specific infectious organism and are linked to a combination of bacterial burden, underlying host immune defenses, preexisting conditions, and the time taken for appropriate intervention. Further prospective randomized controlled trials should identify the specific characteristics of sepsis that contribute to higher mortality rates.
